# Self-directed learning and practice of Italian osteopathic students during summer break: a cross-sectional survey

**DOI:** 10.1186/s12906-019-2619-0

**Published:** 2019-08-06

**Authors:** Giandomenico D’Alessandro, Giacomo Consorti, Francesco Cerritelli

**Affiliations:** 1 Clinical-based Human Research Department, Research Division, COME Collaboration, Pescara, Italy; 2 Research Department of the Centre pour l’Etude, la Recherche et la Diffusion Osteopathiques (C.E.R.D.O.), Rome, Italy; 3 Italian Society of Medical Education (S.I.Pe.M - Società Italiana di Pedagogia Medica), Rome, Italy

**Keywords:** Osteopathy, Osteopathic curriculum, Osteopathic education, Osteopathic students, SDL, SDP, Self-directed learning, Self- directed practice

## Abstract

**Background:**

Self-directed learning is a student-centered approach based on the students’ evaluation about their own learning needs. Self-directed practice is a component of the self-directed learning which focus on achieving manual and practical skills. Given the abundance of manual skills that students need to develop in the osteopathic curriculum, self-directed practice has become an attractive methodology. Most of the Italian osteopathic educational institutions recognize two types of educational curriculum: T1, for students without a healthcare background; and T2 for students who are already healthcare professionals. The aim of the present study is to investigate the attitudes of Italian osteopathic students toward self-directed practice during the summer break highlighting the possible differences between T1 and T2 students.

**Methods:**

A web-based closed-ended survey was administered to the students attending one of the Italian osteopathic educational institutions which accepted to participate in the research.

**Results:**

Two hundred sixty-eight students on 2549 participated to the survey. 64.92% of the students reported to have performed self-directed practice during the summer break. The main difficulty encountered by students in approaching self-directed practice was ‘lack of people to exercise with’. Most of the students performed self-directed practice between 1 to 5 h per week. The most common clinical condition encountered was Low Back Pain. The anatomical area most approached was lumbar spine. The most commonly simulated tests were the articular mobility tests. The techniques in which students trained the most were muscle energy techniques. Significant difference was found in self-engagement to the self-directed practice between T1 and T2 students (*p* = 0.026), and in the reasons to perform self-directed practice (*p* = 0.027).

**Conclusions:**

The results of this study could be useful to reveal some aspects of students’ academic education and future clinical practice. Therefore this paper can be a useful tool for the institutions to develop strategies to overcome the reported problems students have towards self-directed practice. For example it should be discussed the possibility for the students to observe some senior osteopaths during their practice or to plan to maintain an open tutored didactic environment during the summer break as an extracurricular activity.

## Background

Connecting adult learning theory with practice is a challenging task for educators in health profession education [1]. There is the need to reframe the adult education considering the learners as active leaders of their own education [2]. In order to learn adults should understand the process of learning and their role in it, should be actively involved in their own education and must take significant responsibility for their learning [3].

Several strategies have been proposed to achieve these goals, including student-centred approaches [4, 5]. Self-directed learning (SDL) is a student-centered [6] approach based on the students’ evaluation about their own learning needs and the means to satisfy them [7]. SDL is a strategy that potentially develops the skills required to effectively address clinical problems [8] facilitating the acquisition and consolidation of new knowledge and providing attractive social effects for the learners (e.g., cooperation and team building) [6, 9]. SDL in clinical skills can provide an opportunity to build confidence and to improve techniques [10]. Self-directed practice (SDP) is a component of the SDL which focus on achieving manual and technical skills [11]. However, students, might be conditioned by the previous teacher-directed learning experiences and might not be prepared for SDL, leading to difficulty in adapting to SDL [12].

Osteopathy is a primary contact healthcare profession [13], characterized by manipulative techniques, coupling medical and scientific knowledge [14], used to promote patients’ health [15–20]. Osteopaths must be able to perform an accurate and appropriate examination, including relevant clinical testing, observation and palpation to elicit all relevant signs [15]. Osteopathic manipulative treatment (OMT), which follows the osteopathic assessment, currently encompasses a wide range of manual techniques (e.g., balanced ligamentous tension, articulatory, counterstrain, facilitated positional release, high velocity low amplitude and muscle energy) [16, 21].

Given the abundance of manual techniques in the osteopathic curriculum, SDP might be a useful methodology to address the students’ needs regarding the development of technical skills. Furthermore, SDP grants the possibility to accumulate experience. Although the factors that cause large individual differences in professional achievement are only partially understood [22] accumulating experience during the curriculum is important considering that it has been proposed that 10,000 h of deliberate practice might be needed to become an expert in a given field [23]. Indeed, great importance is attributed to offering opportunities for students to practice the physical examination skills extensively [24–27]. Simulation training among medical students is most effective with repetitive practice and self-reflection on performance [28, 29]. Hence autonomous practice when students are not busy following lectures and have more spare time to spend practicing (e.g, during school break) could contribute to gain palpatory and clinical experiences. In turn, this might contribute to raise the perceived preparedness and competence of students which play a role in a potential stressful transition from school to professional practice [30, 31].

Investigation on the autonomous practice of students during the school break with particular regard to US osteopathic curriculum has been made [32]. However osteopathic curricula, may differ depending on countries’ national laws. Indeed differently from the study of Pierce-Talsma et al. [32], the present study has been designed taking into account the Italian curriculum setting which follows the European Committee for Standardization norm (CEN) EN16686 [33]. CEN norm kept the curriculum organization proposed in the “Benchmarks for Training in Osteopathy” [34] recognizing two types of educational curriculum: type I (T1), where students may enter a programme without a healthcare background; and type II (T2) for students who have prior training as healthcare professionals. Prior to the CEN norm [33], T2 curriculum was both for students who have a previous healthcare degree, and who have a previous sport science degree.

Recently, with the law 3/2018 osteopathy became an health profession in Italy [35], whereby osteopathic education may move from the private college system, as it is now, to the higher education sector. With this perspective, it could be useful to collect data about the learning strategies of Italian osteopathic students. To the best of our knowledge there are no studies taking into account the perspective on SDP during summer break of Italian osteopathic students. The aim of the present study is to investigate the features and perceived usefulness of SDP during the summer break of Italian osteopathic students highlighting the possible differences between T1 and T2 curricula.

## Methods

### General information

The study protocol was written following the SURGE reporting guidelines for survey research [36].

For the present descriptive cross-sectional study it have been recruited students from Italian osteopathic educational institutions which accepted to participate in the research. All school were members of the Italian Association of Osteopathic Schools (AISO). A web-based closed-ended survey was used to gather data. Inclusion criteria were (i) to be a student at the second year of the curriculum or above from one of the participant schools;; (ii) to accept the informed consent given before the completion of the survey.

Participation was voluntary and anonymous. Consent to participate was requested before the administration of the survey. Given the online feature of the survey, in order to be able to participate, all respondents had to check a specific box to confirm they had read and understood all the information in the introduction page.

Institutional Review Board of all the participant institutions approved the study.

### Survey development

The survey was developed following three phases: [1] items development; [2] items implementation and survey production; [3] external evaluation.

In the first phase, two researchers (GDA, GC) independently developed a list of items in Italian language which were considered significant in order to investigate the students’ attitudes towards SDP during school summer break. The items generation started by the critical appraisal of similar studies in osteopathic education field [32, 37]. According to the study protocol researchers had to tailor the items used in the other studies to be suitable for the italian frame and to add new ones if needed. In the second phase the researchers critically discussed the usefulness of identified items and selected the items to compose the survey. At the end of the second phase, the questionnaire was composed by two sections: 1) general information section and 2) self-directed practice information (appendix 1). At that point of the survey development, the questionnaire was composed by a total of 32 items, respectively 6 in the first section and 26 in the second section. In the third phase, a third researcher (FC) evaluated the coherence of the items and the face validity of the questionnaire proposing variations where necessary. After the third phase in which a critical debate among the three researchers has carried out, the total number of items was 26, respectively 6 in the first section, and 20 in the second one. 6 items have been removed from the second section because considered redundant or superfluous taking into account the study aim. To allow a coherent answer for the selected question, four types of questions have been used: multiple choices with single possible answer, multiple choices with multiple possible answers, dichotomous and 5-grades likert scale.

### Survey administration and timeline

**Fig. 1:**
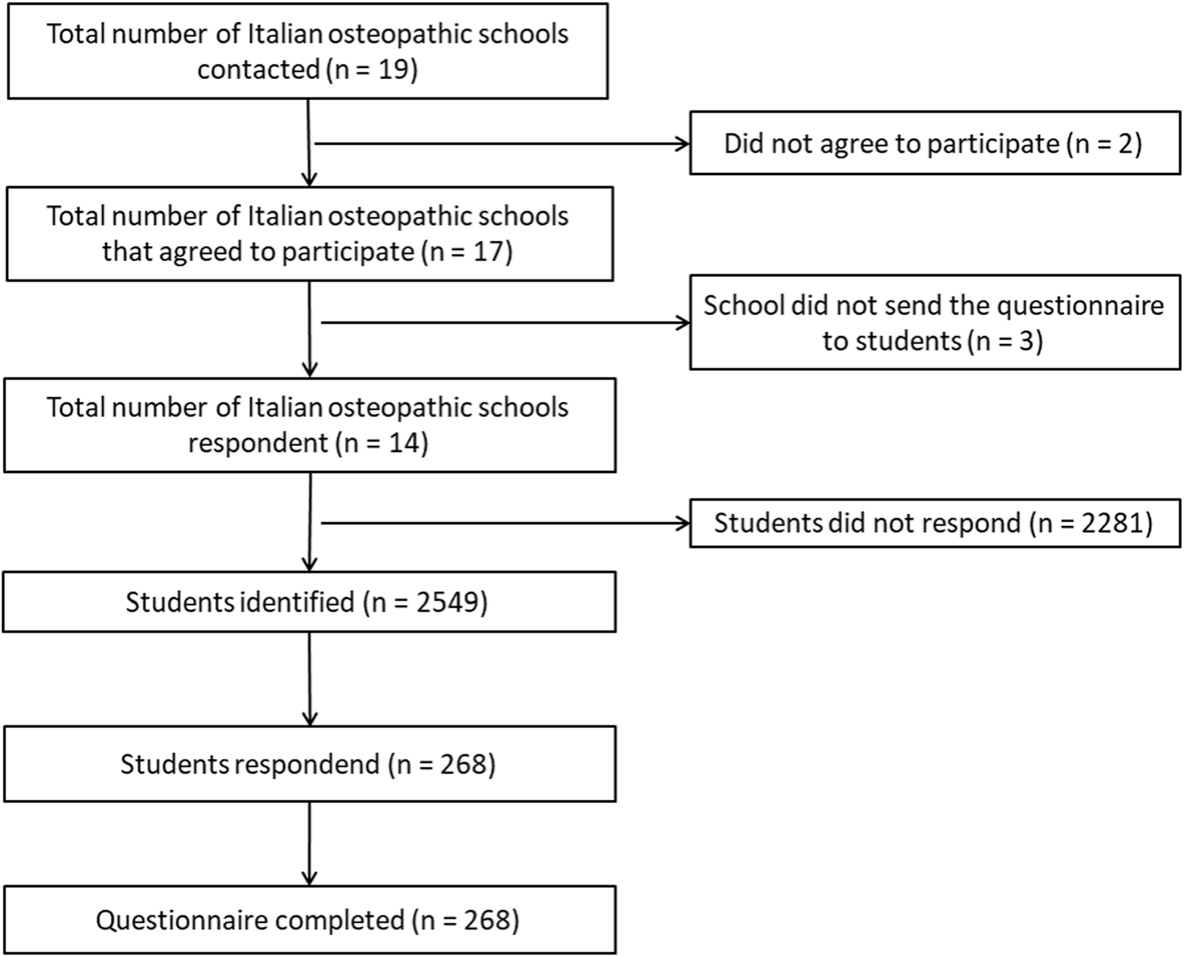
Flow chart of the study

An online survey was administered to all students attending the T1 and T2 programme after brief presentation of the study protocol. The sample was selected among Italian osteopathy students that performed SDP in the previous summer break. On this basis, students of the first year were not included. As in the Pierce-Talsma et al. study, students were not informed before the summer break that the survey would be distributed [32] to avoid any change of SDP practice. The survey was accessible for three weeks after the students returned from the summer break, from the 11st September 2017 to the 1st October 2017, throughout a dedicated and protected weblink. According to the study protocol two reminders for participation were sent by e-mail to students. Timeline of all processes has been summarized in Fig. 1.

### Data collection and statistical analysis

Anonymous data were collected throughout an ad-hoc platform developed by the Center for Osteopathic Medicine (COME) Collaboration and successfully previously tested. Data were stored on a dedicated server which was accessible only by the data manager. The system automatically managed the possibility of double respondents by impeding two or more accesses to the survey from the same e-mail address if it had already completed the survey. A codebook was used to convert the data. After the data collection phase, data were exported, mined and processed by an independent statistician who was blinded to study protocol.

Results were expressed in terms of point estimate and 95% of confidence interval (CI). Arithmetic mean, standard deviation, percentage, range, median and interquartile range were used. Binary data was compared using the chi square test, continuous data was tested using Student t test. Significance levels were set at an alpha level less than 0.05.

## Results

### Sample

The study protocol was initially presented to all member schools of AISO (*n* = 19) and 17 of theseagreed to participate. Among these 17 schools 14 (82.35%) effectively sent the web-link to access to the survey to the students. A total of 268 students participated to the survey showing a response rate of 10.51% (268/2549 e-mails sent). All of the respondents completed the survey (completion rate = 100%; *n* = 268/268). The mean age was 27.69 (5.52); the males were138(51%).

Considering T1 and T2 groups, data showed statistically significant differences for gender (*p* = 0.005), age (*p* < 0.001), year of course distribution (*p* = 0.01). Additionally the T2 programme had a significant higher number of students with a previous healthcare degree than T1 (50.6% vs 2%, *p* < 0.001) and a higher number of offsite students than T1 (55.36% vs 28%, *p* < 0.001). Full data of the surveyed sample are presented in Table 1.

**Table 1:** Sample

	T1	T2	p
Total	100	168	
Gender	F = 60 (60%)	F = 70 (41.67%)	0.005
	M = 40 (40%)	M = 98 (58.33%)	
Age*	23.48 ± 2.98	30.20 ± 5.14	< 0.001
Year of frequention before summer break	4°: 26 (26%)	3°: 33 (19.64%)	0.01
	1°: 24 (24%)	4°: 32 (19.02%)	
	2°: 20 (20%)	2°: 28 (16.67%)	
	3°: 14 (14%)	5°: 27 (16.08%)	
	5°: 14 (14%)	1°: 25 (14.89%)	
	6°: 2 (2.00%)	6°: 23 (13.70%)	
Previous healthcare degree	2 (2%)	85 (50.6%)	< 0.001
Transfer students	28 (28%)	93 (55.36%)	< 0.001

Data are presented as N (%) and *mean (sd). *P* values from chi square test or *student t test. T1 = Type 1; T2 = Type 2

### Attitudes of the students towards SDP

Considering the whole sample, results showed: 94/268 students (35.08%) did not perform at all SDP during the summer break. The reported reasons were ‘lack of appropriate place’ (42/94, 44.68%), ‘lack of subjects to exercise with’ (40/94, 42.55%) and ‘lack of time’ (36/94, 38.30%). No students reported that ‘the self-direct practice was useless’.

174/268 students (64.92%) reported to have performed SDP during the summer break and considered it useful (97.13%; *n* = 169).

Ninety-eight respondents out of the 174 who reported to have performed SDP (56.32%) declared not to have sufficiently practised during the period and among these 98 responders almost the totality (*n* = 94; 94.92%) stated they would have practice more SDP and reasons were ‘lack of people to exercise with’ (50/94 = 53.19%), followed by ‘lack of time’ (43/94 = 45.75%) and ‘lack of appropriate location’ (38 out of 94 = 40.43%).

Students performed SDP for an amount of time spawning between 1 to 5 h per week (59.20%; *n* = 103).

The most frequent difficulty was ‘the interpretation of osteopathic tests’ (*n* = 164; 94.25%); the less frequent one was ‘the relationship with patients’ (*n* = 84 = 48.28%).

When the students were in difficulty they seeked assistance mostly from ‘a peer’ (27.35%) followed by ‘a more expert osteopath’ (21.80%), ‘a teacher’ (9.83%), ‘a senior student’ (7.69%), ‘an healthcare professional’ (7.26%). A quarter of students (26.07%) did not ask for any help.

Regarding SDP evaluation the 69.54% of respondent students (*n* = 121) evaluated the level of their SDP using more often a self-evaluation method (73.55%; *n* = 89) rather than other methods such as peer feedback (14.05%; *n* = 17) expert student feedback (6.61%; n = 8), expert osteopath (5.79%; *n* = 7).

The 46.55% (*n* = 81) reported a decrease in their SDP during the summer break; the 32.18% (*n* = 56) reported an increase in their SDP and the 21.27% (*n* = 37) reported no changes in the frequency their self-reported activity compared to the rest of the year.Most of the students on (70.16%; *n* = 136) performed SDP at parents or friends’ studio.

The most common clinical condition encountered by students was Low Back Pain. The anatomical area most approached by students was lumbar spine. The most commonly simulated tests were the articular mobility tests. The techniques in which students trained the most were muscle energy techniques. An extensive summary of the clinical conditions, anatomical areas, tests and techniques are available in figures from 2 to 5.

### Differences between T1 and T2

Considering the difference between the T1 and T2 group (Table 2), a further analysis showed a statistically significant difference in self-engagement to the SDP (T1: *n* = 56; 56.00% vs T2: *n* = 118; 70.24%; *p* = 0.026), and in the reasons to perform SDP (*p* = 0.027). T2 students performed self-directed practice mostly because they aimed to reproduce a situation they would have met in the future professional practice (T2 = 42.86% vs T1 = 37.36%), whereas T1 students approached SDP mostly for practic purpose (T1 = 38.78% vs T2 = 21.69%).

**Table 2:** Self-directed practice between T1 and T2

	T1	T2	p
Self-directed practice	Yes 56 (56%)	Yes 118 (70.24%)	0.026
	No 44 (44%)	No 50 (29.76%)	
Why?	Practice 38 (38.78%)	Future clinic work 81 (42.86%)	0.027
	Future clinic work 37 (37.76%)	Practice 41 (21.69%)	
	Exam 12 (12.24%)	Exam 40 (21.16%)	
	Not enough practice during academic year 7 (7.14%)	Not enough practice during academic year 14 (7.41%)	
	Other 4 (4.08%)	Other 13 (6.88%)	
Why not? (Lack of …)	Appropriate place 21 (27.27%)	Practice buddies 22 (25.00%)	0.247
	Practice buddies 18 (23.38%)	Time 21 (23.86%)	
	Time 15 (19.48%)	Appropriate place 21 (23.86%)	
	Safety 10 (12.99%)	Other 14 (15.91%)	
	Other 7 (9.09%)	Safety 9 (10.23%)	
	Motivation 6 (7.79%)	Motivation 1 (1.14%)	
	Usefulness 0 (0.00%)	Usefulness 0 (0.00%)	
Hours of self-directed practice/week	1–5: 39 (69.64%)	1–5: 64 (54.24%)	0.261
	6–10: 9 (16.07%)	6–10: 27 (22.88%)	
	11–15: 5 (8.92%)	11–15: 14 (11.86%)	
	> 16: 3 (5.37%)	> 16: 13 (11.02%)	
Practice compared to the rest of the year	diminished: 32 (57.14%)	diminished: 49 (41.52%)	0.150
	increased: 15 (26.79%)	increased: 41 (34.75%)	
	unvaried: 9 (16.07%)	unvaried: 28 (23.73%)	
Clinic simulation	sometimes: 21 (37.5%)	sometimes: 38 (32.2%)	0.26
	never: 12 (21.43%)	often: 28 (23.73%)	
	rarely: 11 (19.64%)	rarely: 19 (16.11%)	
	often: 10 (17.86%)	never: 18 (15.25%)	
	always: 2 (3.57%)	always: 15 (12.71%)	

T1 = Data are presented as N (%). *P* values from chi square test. T1 = Type 1; T2 = Type 2

## Discussion

This study investigated the features and perceived usefulness of SDP during the summer break of Italian osteopathic students highlighting the possible differences between T1 and T2 curricula. A better understanding of the features and perceived usefulness of SDP of osteopathy students is critical to identify the needs of students to lead possible improvements in the academic curriculum [32].

Demographic data showed a trend of T1 students being younger than T2. The reason could be the beginning of osteopathic education soon after the end of the high school for T1 students. By contrast, T2 students enter the osteopathic school after the completion of an additional healthcare degree. Interestingly, T1 and T2 have an opposite distribution in gender ratio: T1 has more female students. The study of Bleakley highlights a “feminising trend” in medicine. Indeed, women are the majority in terms of entry to medical schools worldwide [38]. This trend is confirmed in the present study only considering T1 students.

Results of the present study showed that T1 students performed less SDP than T2 during the summer break. This significant result could be related to the fact that T1 might not engage in SDP over summer as they have likely not been required to before entering their osteopathy course. Previous exposure to study at an adult education level may help develop SDL behaviours. Furthermore, T2 students are usually already working professionals so they might have a students are usually already working professionals so they might have a perspective oriented to life-long learning [39]. That hypothesis is supported by the significant difference between the reasons for which T1 and T2 students performed SDP. In particular, T2 students reported to perform SDP to be prepared for the future clinical practice. T1 students seem to engage SDP both to develop technical and manual skills and to the be prepared for the future clinical practice. According to one assumption in pedagogy “*assessment drives learning*” [40], T2 students reported to have practiced SDP in view of future exams. The findings of the present study are consistent with the study of McGrath et al. [8] which demonstrated that imminent clinical skills examinations appear to incentivise engagement with SDP. One of the possible explanation of the observed difference between T1 and T2 could be, as shown by Deng et al. [41] that students engage extensively in SDP and that it is associated with superior performance on a medical licensing examination.

**Fig. 2:**
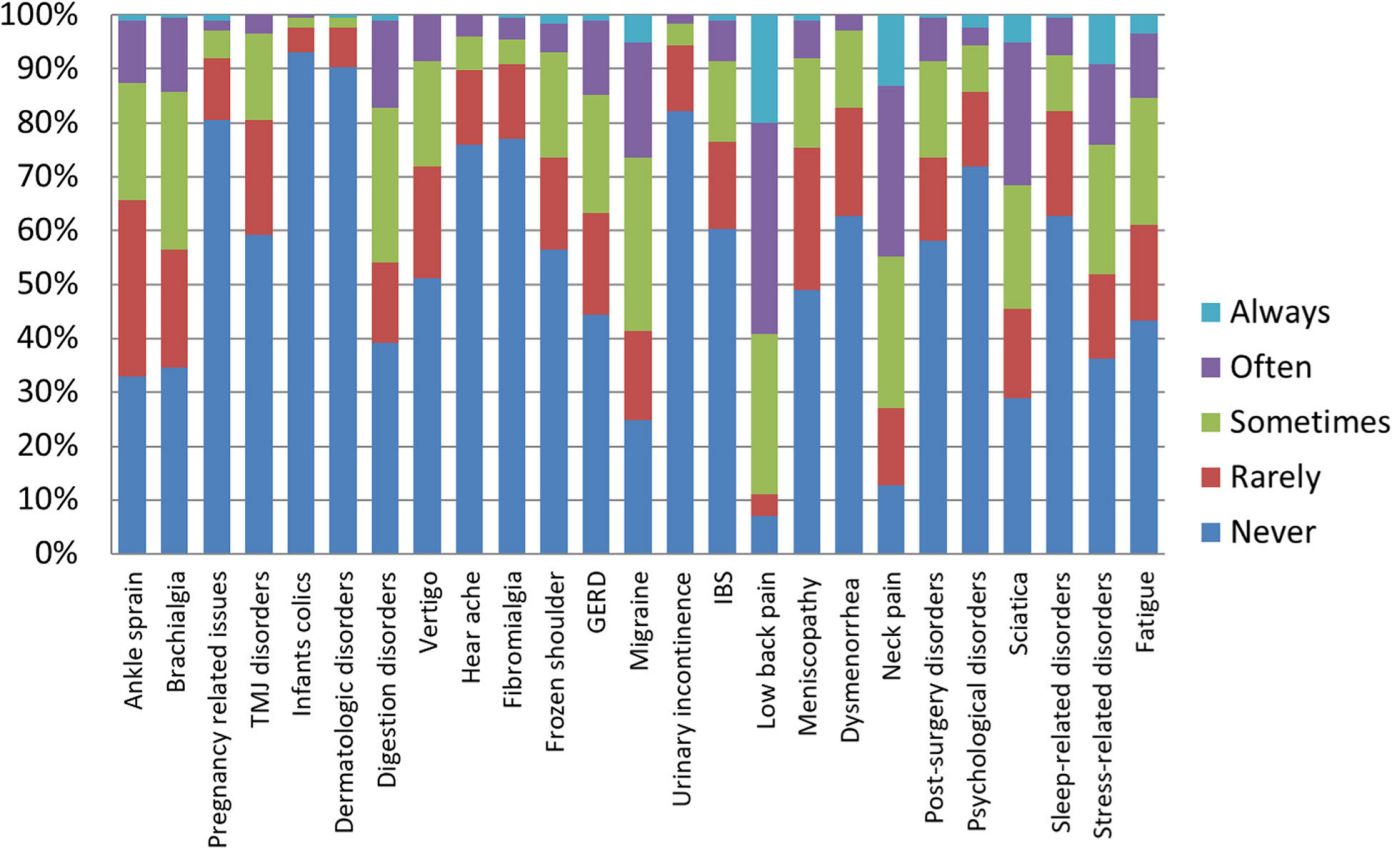
*«How often did you engage self-directed practice on practice buddies with the following conditions?»*. Students had to choose one answer (Always, Often, Sometimes, Rarely, Never) for each of the 25 clinical conditions listed. The most common clinical condition encountered was ‘Low back pain’. The less common clinical condition encountered was ‘Infants colics’. TMJ: Temporo-Mandibular Joint; GERD: Gastroesophageal reflux disease; IBS: Irritable Bowel Syndrome

**Fig. 3:**
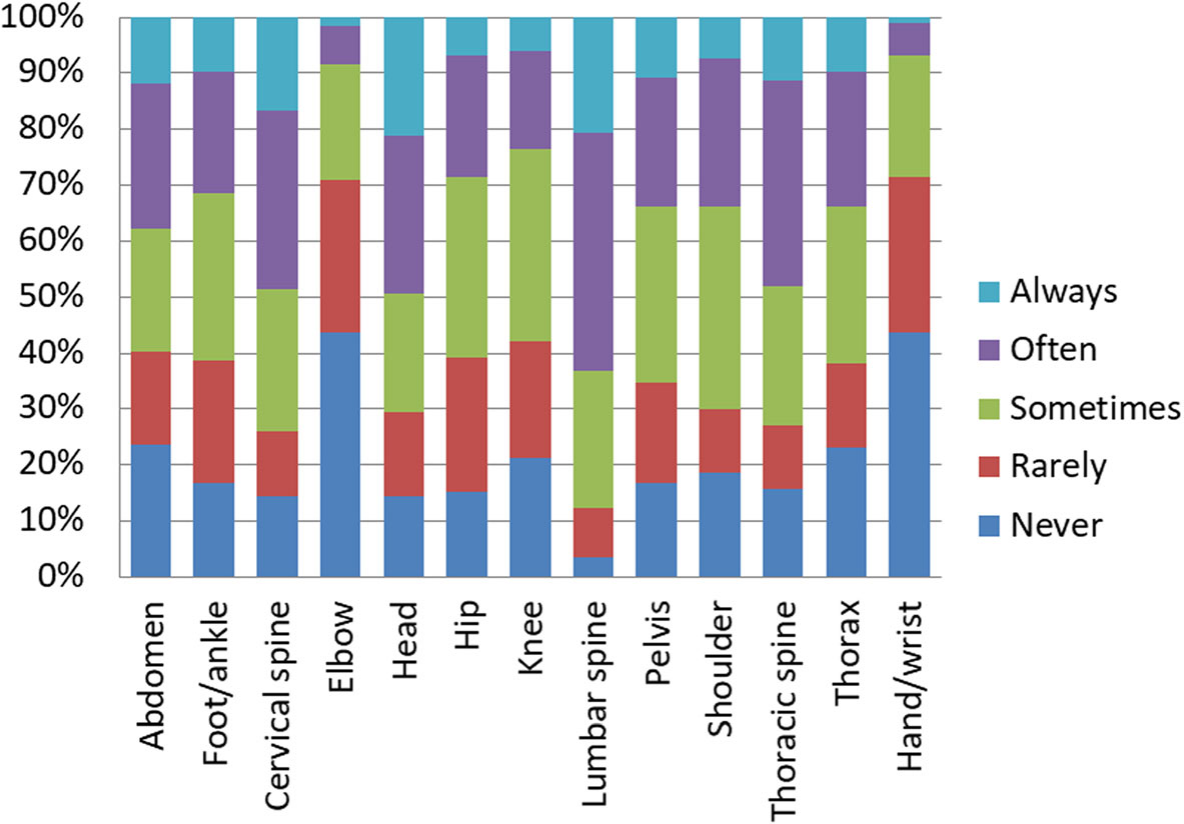
*«How often did you engage self-directed practice on the following body areas?»*. Students had to select one answer (Always, Often, Sometimes, Rarely, Never) for each of the 13 body areas listed. The most common area approached by students was ‘Lumbar Spine’. The less common area was ‘Hand/Wrist’

**Fig. 4:**
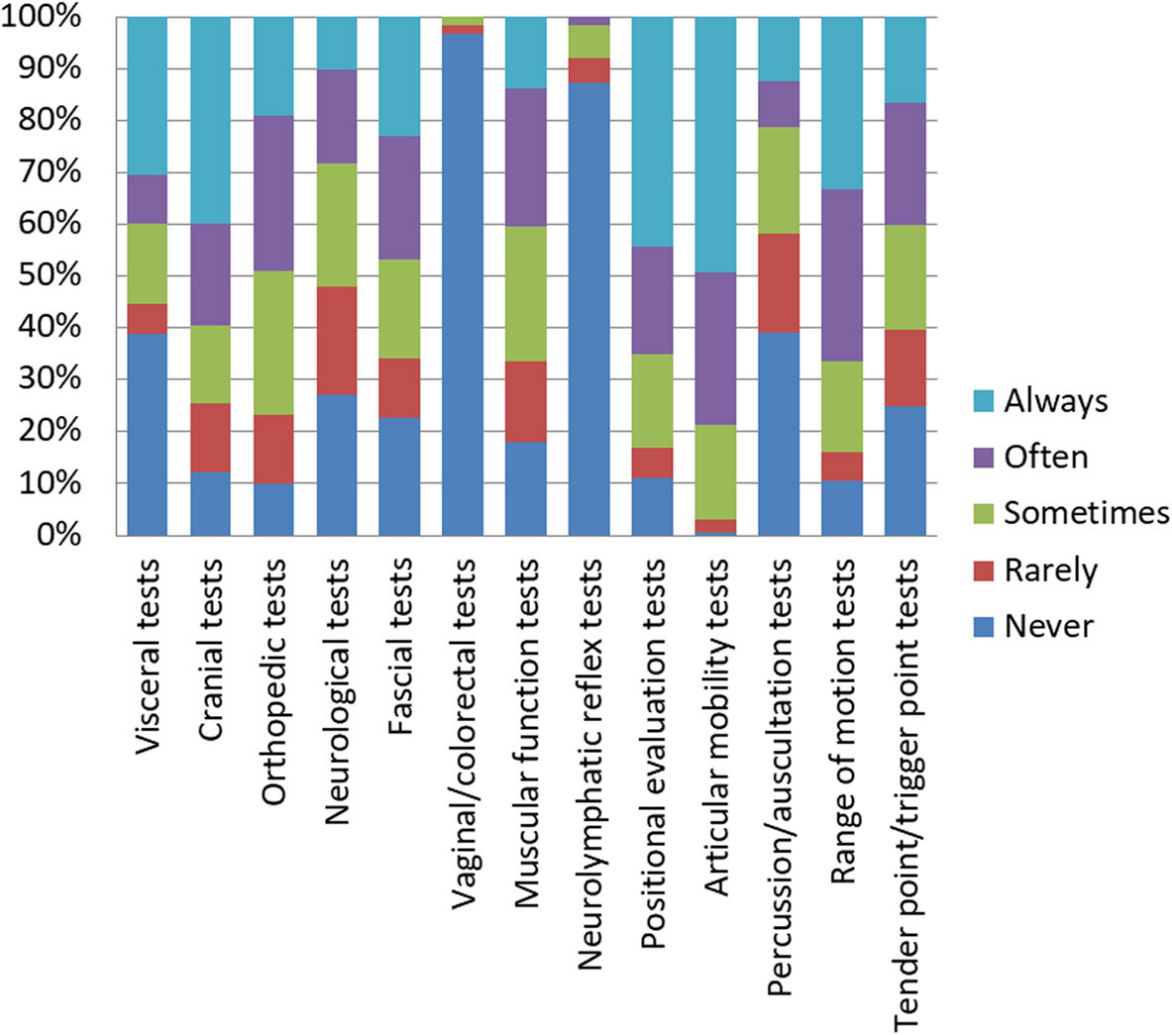
*«*How often did you engaged self-directed practice on the following tests?*»*. Students had to choose one answer (Always, Often, Sometimes, Rarely, Never) for each of the 13 tests listed. The most common used test was ‘articular mobility test’. The less used test was ‘Neurolymphatic reflex tests’

**Fig. 5:**
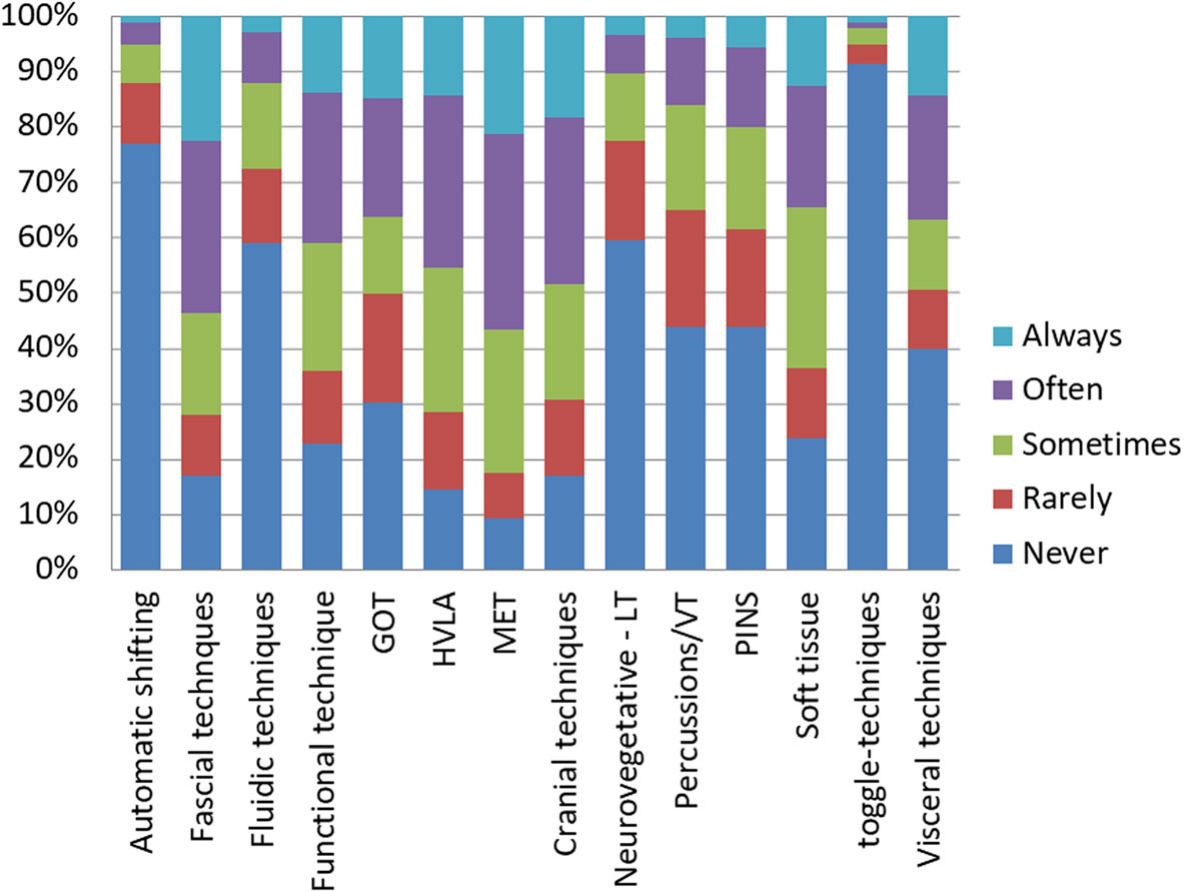
*«*How often did you engaged self-directed practice on the following techniques?*»*. Students had to select one answer (Always, Often, Sometimes, Rarely, Never) for each of the 14 techniques listed. The most used technique was Muscle Energy Technique (MET). The less used technique one was ‘Toggle-techniques’. GOT: General Osteopathic Treatment; HVLA: High Velocity Low Amplitude; LT: lymphatic techniques; VT: vibrational techniques; PINS: progressive inhibition of neuromuscular structures

Almost the totality of the students who practiced the SDP during summer break consider it useful, but more than half of them reported that the amount of SDP was not sufficient since they would have like to perform it more. The reasons why students did not perform or did not perform sufficiently SDP should be considered to find appropriate solutions. The main difficulties varies from personal and general reasons: lack of time, lack of an appropriate place, lack of time and practice buddies. These factors could be potentially challenged through an active strategies that involves both students and schools. One of the reasons which prevented the students to practice SDP during the summer break was the lack of a dedicated didactic/clinical environment. This can be a useful data for the schools to plan to maintain an open tutored didactic environment during the summer break as an extracurricular activity. It may be interesting to evaluate the attitudes of students towards the SDP during the extracurricular activities and to assess if it produces as a complementary effect an increase of students’ entrepreneurial behaviour towards their future profession. Furthermore it is possible for the osteopathic institutions to suggest students to organise some autonomous meetings during the summer break to overcome the reported lack of practice buddies. Pierce-Talsma et al. [32] suggested the possibility to enhance in the curriculum those techniques that do not require a table and that require less time to challenge the lack, respectively, of appropriate place and time. Furthermore, the institutions might potentially make space available to students, allowing them the physical space to practice and practice with peers. To evaluate if SDP is aligned to the future clinical scenario it is possible to compare the clinical conditions encountered at least once by students (Fig. 2) and those encountered by professional osteopaths [42]. There is a quite overlapping between them. Indeed low back pain, neck pain, headache, sciatica are the most common clinical conditions faced both from students and from professionals. It is possible to consider that self-directed practice starts to show clinical reality. The students, in this way, could begin to interact with common elements of those so frequent conditions to be met in the future. The lumbar and cervical spine, skull and pelvis were the most common anatomical areas on which students practiced (Fig. 3). This trend is similar to the one found by van Dun et al. [20]. It may be interesting in future studies to qualitatively evaluate the reasons of this prevalence. A possible explanation is that techniques on these areas are perceived by students as more difficult and therefore they need more practice to be mastered. Another possible explanation is related to the idea that students may have of the professional identity. It is possible that they look at the osteopathic professional as the healthcare professional that mostly work on these zones. Since part of the professional identity is learned by students through hidden curriculum [43] understanding the reasons underlying the prevalence of SDP per area could allow the institutions to start a curricular improvement process and faculty development activities [44, 45]. In the present study ‘articular mobility test’, orthopedic test’ and ‘positional evaluation test’ were the most practiced tests (Fig. 4). This finding is only partially comparable to the one reported by Van Dun et al. [20]. Indeed, in their study the most used tests were ‘inspection’, ‘positional evaluation test’ and ‘articular mobility test’. The biggest discrepancy with the Van Dun et al. study regards the most used techniques. Students from the present study reported a frequent use of the following techniques (Fig. 5): ‘muscular energy techniques’, ‘high velocity low amplitude techniques’ and ‘fascial techniques’, while professionals from Van Dun et al. study declared to use more frequently: ‘visceral manipulation’, ‘cranial techniques’ and ‘general osteopathic mobilization’ [20]. This discrepancy might depend on the fact that respondents of Van Dun et al. study were professional osteopaths while in the present study respondents were osteopathy students.

A major students’ concern emerged from the survey was related to the interpretation of the osteopathic manual tests (94.25%; *n* = 164). The heterogeneity of the interpretation of a palpatory osteopathic test is a central topic in osteopathic literature as highlighted by Basile et al. [46]. The literature shows that students’ monitorization of their own learning is important in self-directed learning [47]. Indeed, it is critical for learners to monitor their learning in line with their own needs and goals, to identify wrong or deficient learning and to adopt new strategies accordingly [48]. It is possible that students might not be able to develop their own learning goals as they do not always know the most appropriate level at which to pitch the goal. Furthermore, a possible way to manage these deficiencies might be for the institutions to focus on a consensus training methodology during the in-class training. It has been demonstrated that a specific consensus training could positively affect the inter-rater reliability of an osteopathic test [49].

One of the strengths of the present study is the heterogeneity of sample coming from different Italian osteopathic schools. Secondly, the prompt administration of survey soon after the end of summer break potentially reduces the recall bias of student in remembering the features of their SDP.

This study has several limitations. First, the item creation process might have intrinsic bias due to the low number of researchers involved. Despite one of the researchers has a qualification in osteopathic education, it cannot be excluded that researchers own beliefs regarding SDP might have influenced the items creation. Secondly, the low response rate (10.51%). Indeed a similar study conduct in USA achieved a way higher response rate (81.6%) [32]. Thirdly, considering the algorithm of survey administration it is possible that there have been some issues with [1] e-mail sending/receiving process, [2] solicitation to the students, [3] survey return to the database. The low response rate implies another limitation: a low sample size that impedes to do different sub analyses and this could potentially prevent statistical significance in some results. Furthermore it is possible that the sample may not be representative of the actual population of osteopathic students in Italy. This aspect should be control in the future studies.

## Conclusion

This study is a cross sectional survey aimed to investigate the features and perceived usefulness of SDP during the summer break of Italian osteopathic students. Despite some different findings between T1 and T2 students like inclination to SDP and reasons why they perform it, the entire sample shown an homogeneous trend regarding the majority of the aspects of SDP. Findings of this study are consistent with the available medical education literature. The results of this study might be useful to reveal some aspects of students’ academic education and future clinical practice. Despite the number of respondents is currently too low to draw any specific conclusions considering the higher education of Osteopathy in Italy, the present findings might be useful information to tailor implementation of SDL to the students’ needs. Therefore, to develop strategies to overcome the reported problems students have towards SDP this survey-based study is potentially replicable in the years to have a perspective view on the evolution of summer break SDP on a larger sample.

## Data Availability

All relevant data are within the paper.

## Abbreviations

CEN: European Committee for Standardization norm
OMT: Osteopathic manipulative treatment
SDL: Self-directed learning
SDL: Self-directed practice
T1: Type 1
T2: Type 2
